# Atazanavir versus lopinavir on Covid-19 infection: A retrospective protease inhibitors comparative study 2020

**DOI:** 10.22088/cjim.13.0.173

**Published:** 2022

**Authors:** Ahmad Alikhani, Mobin Ghazaiean, Roya Ghasemian, Mohammad Khademloo

**Affiliations:** 1 Department of Infectious Diseases, Antimicrobial Resistance Research Center and Communicable Disease Institute, Mazandaran University of Medical Sciences, Sari, Iran; 2 School of Medicine, Mazandaran University of Medical Sciences, Sari, Iran; 3 Department of Community Medicine, Mazandaran University of Medical Sciences, Sari, Iran

**Keywords:** Atazanavir / ritonavir, lopinavir / ritonavir, Side effects, Covid-19, Efficacy

## Abstract

**Background:**

Evaluation of protease inhibitors (PIs) is important in terms of prescribing an effective regimen for reducing mortality and hospitalization in Covid-19. Therefore, follow-up of patients better determines the characteristics of existing regimens.

**Methods:**

We retrospectively evaluated the demographic, co-morbidities, gastrointestinal (GI) and liver complications of patients at two teaching hospitals from the first of March to the end of July 2020. All patients received one of two recommended regimens including hydroxychloroquine (HCQ) (400 mg BD on the first day and then 200 mg BD) plus atazanavir/ritonavir (ATV) (300/100 mg daily) or HCQ with the same dose plus lopinavir/ritonavir (Kaletra) (400/100 mg BD) for 5-7 days.

**Results:**

We chose 170 cases that received 2 different regimens. In group one, 85(57.6% males) patients received Kaletra and HCQ and group two, 85 (55.3% males) patients received ATV and HCQ. The study of hospitalization in both groups showed no difference in more or less than 5 days hospitalization. (P=0.757) Comparison of mortality rates has not shown a significant difference including 19 (22.4%) deaths in group 1 and 15(17.6%) deaths in group 2 (P=0.443). Nausea followed by diarrhea was the most common side effects in group 1. But no side effects were reported in group 2 (P=0.000). Abnormal liver function tests (LFTs) were seen in both groups.

**Conclusion:**

Comparison of hospitalization and mortality were not statistically significant. It seems that a respect to similar effect on mortality and hospitalization. ATV regimen is superior to Kaletra especially for better GI tolerance and less daily pills.

Various medications have been tested to find Covid-19 cure but none of them had efficiency approved.(1) On the other hand, World Health Organization (WHO) recommended symptom management due to lack of approved cure. National health commission (NHC) suggested several antiviral agents such as chloroquine phosphate (CQ), Kaletra, interferon, arbidol and ribavirin in the latest guidelines. (2) It has been demonstrated that the most efficient antiviral agents were those which invaded viral enzymes. Among those agents, Kaletra acts against major protease of the virus (Mpro), while CQ and interferon (IFN)-β invade host cells directly.(2, 3) About 2000 Kaletra has been replaced by PI agent due to lower side effects called ATV.(4, 5) It had shown better activity against Mpro.(6) It inhibits the enzyme that has a role in viral gag processing which is developed to non-infected viruses.(7) Now, it is necessary to identify specific and effective antiviral therapies for Covid-19.(8, 9) We decided to evaluate the safety and efficacy of HCQ and ATV regimen versus HCQ and Kaletra regimen in a retrospective study.

## Methods

**Study design and participants: **170 patients admitted in two teaching hospitals suspected for Covid-19 and had positive polymerase chain reaction (PCR) test (samples obtained from nasopharyngeal and oropharyngeal) or lung computed tomography (CT) scan compatible to Covid-19 entered to the study. In this retrospective study, two different combination regimens were compared, Kaletra+HCQ and ATV+HCQ. The Ethics Committee of Mazandaran University of Medical sciences approved the study (approval ID:IR.MAZUMS..REC.1399.8688).

**Clinical method and assessment of clinical** **parameters: **In this retrospective cohort study, two groups of 85 patients were isolated from the hospitalized Covid-19 patients who did not have a severe form of the disease. Thus, for the groups, first HCQ was started, then, one group was given ATV diet and the other group was given Kaletra regimen. The regimen of group 1 included Kaletra (400mg / 100mg) every 12 hours. Patients of group 2 took ATV (300 mg + 100 mg) once daily. However, both groups received 400 mg HCQ tablets for 7 days.

All patients were enrolled at baseline with 90-94% O2 saturation (O2sat). In fact, they were not of the severe type, because they had an O2 sat above 90% at the time of admission(10). Patients who initially had an O2 sat above 90% and gradually developed below 90%, or other adjunctive drugs were started like dexamethasone, methylprednisolone, IFN was excluded. Also, another treatment modality was started for patients who initially had less than 90% O2 sat. As a result, these two groups were excluded from the study. On the other hand, according to the findings of the patients’ CT scan, all patients admitted to the study had less than 50% involvement.

Patients in both groups were surveyed in terms of demographic findings(age and sex), hospitalization, mortality rates, LFTs that included alanine aminotransferase (ALT), aspartate aminotransferase (AST) and alkaline phosphatase* (*ALP) before and after regimens, co-morbidities[including diabetes mellitus (DM), hypertension (HTN), heart failure (HF), chronic kidney disease(CKD), chronic liver disease (CLD), ischemic heart disease(IHD), dyslipidemia(DLP), asthma and hypothyroidism], plus GI complications(such as nausea, vomiting, abdominal pain, epigastric pain, bloody vomiting , reflux and diarrhea) while receiving antiviral regimens. In patients whose Kaletra regimen caused mild GI upset, the side effects were treated symptomatically and Kaletra was maintained. In 4 patients with severe side effects Kaletra was changed to ATV for the remaining days. 

**Data collection:** Two physicians independently recorded demographic information, co-morbidities, GI and liver complications of antiviral regimens, mortality rates, and hospitalization.

**Outcomes: **The primary outcome encompassed the comparison of mortality rates between the two groups. Secondary outcome included the length of hospital stay, GI and liver side effects.

**Statistical analysis: **Comparative data analysis between the two groups was performed with SPSS Version 22 (IBM SPSS Statistics). Also, data were reported with median n%. Qualitative data including demographic information, co-morbidities, hospitalization and mortality data, and GI complications were compared between Kaletra and ATV groups. Qualitative variables of the two groups were evaluated using chi-square test. For all variables, p<0.05 was considered significant.

## Results

170 patients received two different antiviral regimens. 88(51.8%) and 848.2%2) patients were over and under 65 years old, respectively. Patients participating in the study were divided into three age groups. In both groups, the age over 65 years was more common and among the patients under 65 years, more of them were in ATV group. 43.5% of the patients in the study were women, but there was no significant difference in terms of female sex hospitalization between the two groups.

HTN, IHD, and DLP were the most common in patients of the Kaletra group. On the other hand, HTN and DM were the most common co-morbidities in group 2. Eighty percent of hospitalized patients were complicated with one of the cardiovascular diseases (such as IHD, HF or HTN), which was about 2.5 times in group 1 compared to the ATV group. About half of the patients in the study had HTN (the number of patients in group 1 was about 2.5 times higher than in group 2) (P=0.000). About one-sixth of the patients in the study had a history of DM, which was 3.5 times more common in group 2 than in group 1. (P=0.002). The detailed demographic and co-morbidities profile data of all patients were summarized in table 1.

We showed the length of hospital stay with cutoff point of 5 days and mortality rates of 19(22.4%) in group 1 and 15(17.6%) in group 2, respectively, which are mentioned in detail in table 2. Both p-values were greater than 0.05 between the two groups. 56.5% of patients had ≥5 days of hospitalization in which no significant differences were seen between patients of the two groups (P=0.757). Furthermore, 20% of patients died during the study, which was higher in group 1 but was not statistically significant (P=0.443) (table 2). The overall mortality rates by sex predominance in the two groups demonstrated that among the 34(20%) patients who died, 26(27.1%) were males and 8(10.8%) were females (P=0.009). The questionnaire of GI complications was completed at the same time as patients received combined antiviral regimens in both groups. All this information was summarized in table 3. GI side effects were not seen with ATV regimen, while nausea 28(16.5%) and diarrhea 23(13.5%) were the most common ones in Kaletra group. Meanwhile, abnormal LFT was seen in some patients of both groups (table 3).

**Table 1:** Demographic and co-morbidities data of the study patients

Findings	Total N=170	ATV+HCQ N=85	Kaletra+HCQ N=85	P value
**Age, year**	40(< 50 year) 42(50-65 year) 88(> 65 year)	28(< 50 year) 21(50-65 year) 36(> 65 year)	12(< 50 year) 21(50-65 year) 52(> 65 year)	0.010
**Female sex**	74(43.5%)	38(44.7%)	36(42.4%)	0.757
**Co-morbidities**				
**Diabetes mellitus**	27(15.9%)	21(24.7%)	6(7.1%)	0.002
**Hypertension**	86(50.6%)	24(28.2%)	62(72.9%)	0.000
**Heart failure**	13(7.6%)	10(11.8%)	3(3.5%)	0.043
**Chronic kidney disease**	7(4.1%)	6(7.1%)	1(1.2%)	0.054
**Chronic liver disease**	3(1.8%)	3(3.5%)	0(0.0%)	0.081
**Ischemic heart disease**	37(21.8%)	5(5.9%)	32(37.6%)	0.000
**Dyslipidemia**	30(17.6%)	0(0.0%)	30(35.3%)	0.000
**Asthma**	4(2.4%)	4(2.4%)	0(0.0%)	0.043
**Hypothyroidism**	1(0.6%)	0(0.0%)	1(1.2%)	0.316

ATV: atazanavir, HCQ: hydroxychloroquine,

**Table 2:** Stay of hospitalization and mortality rates findings

Findings	Total N=170 N (%)	ATV+HCQ N=85 N (%)	Kaletra+HCQ N=85 N (%)	P value
**Length of hospitalization** **(< 5 days)** **(≥ 5 days)**	74(43.5%) 96(56.5%)	38(44.7%) 47(55.3%)	36(42.4%) 49(57.6%)	0.757
**Mortality** **Rates of death**	34(20%)	15(17.6%)	19(22.4%)	0.443

ATV: atazanavir, HCQ: hydroxychloroquine,

**Table 3:** Gastrointestinal complications findings

Findings	Total N=170	ATV+HCQ N=85	Kaletra+HCQ N=85	P value
**Gastrointestinal side effects**				
**Nausea**	28(16.5%)	0(0.0%)	28(32.9%)	0.000
**Vomiting**	12(7.1%)	0(0.0%)	12(14.1%)	0.000
**Epigastric pain**	20(11.8%)	0(0.0%)	20(23.5%)	0.000
**Abdominal pain**	17(10.0%)	0(0.0%)	17(20%)	0.000
**Bloody vomiting**	0(0.0%)	0(0.0%)	0(0.0%)	
**Reflux**	6(3.5%)	0(0.0%)	6(7.1%)	0.013
**Diarrhea**	23(13.5%)	0(0.0%)	23(27.1%)	0.000
**Liver function test**				
**Elevated ALT** **(NL limit < 45 U/L)**	6(3.5%)	3(3.5%)	3(3.5%)	1.000
**Elevated AST** **(NL limit < 35 U/L)**	5(2.9%)	3(3.5%)	2(2.4%)	0.650
**Elevated ALP** **(NL limit < 120 U/L)**	6(3.5%)	3(3.5%)	3(3.5%)	1.000

ATV: atazanavir, HCQ: hydroxychloroquine, ALT: alanine aminotransferase, AST: aspartate aminotransferase, ALP: alkaline phosphatase

## Discussion

We found that patients who received Kaletra regimen had relatively similar hospitalization compared to group 2, but GI complications were more commonly reported in the former group (table 3). There was also a higher mortality rate in group1 but it was not statistically significant (table 2). Given the unknown behavior of the coronavirus and the fact that there is no definitive cure for it, researchers are still trying to choose drugs with the least side effects and also cost-effective ones. Antiviral agents have a pivotal influence in the first phases of replication (11, 12). Overall, PIs are the agents that prohibit processing of viral entry through inactivation of envelope glycoproteins (13). Kaletra has already been recommended for the human immunodeficiency virus(HIV) (14). It has been proven that lopinavir (LPV) inhibited the replication of Middle East respiratory syndrome coronavirus (MERS-CoV) and severe acute respiratory syndrome coronavirus (SARS-CoV) (15). Ritonavir (RTV) has no role in viral replication but it has been revealed that RTV affects LPV bioavailability by prohibiting cytochrome P450 3A4 enzyme (16).

There have been number of controversies about the effect of Kaletra on viral clearance. The safety and effectiveness of Kaletra have been reported by some studies but had no impact on mortality (17, 18). In a study of 134 patients who initially received IFN-alpha 2b inhalation; 52 patients received Kaletra, 34 patients received arbidol and 48 patients did not receive any regimen. 

The mean time of negative PCR test (in all 3 groups after 7 days of hospitalization), symptom relief, and the mean time of radiological improvements (after 7 days of hospitalization between the 3 groups) did not show any difference (17). Although the efficacy of Kaletra has been approved for SARS and MERS-CoV, a trial of 199 SARS-CoV-2 patients by Cao B et al. did not show a benefit for clinical improvement, while it showed a sort of efficacy in the early stages of the disease. In a randomized controlled trial (RCT) of 199 participants, 99 patients received Kaletra regimen and 100 patients were control. Patients' findings were compared in terms of clinical symptom relief, and mortality rates. PCR findings of the two groups at different times showed similar findings. The improvement in symptoms indicated only one day earlier in the Kaletra group. Comparison of 28-day mortality between the two groups was not different significantly. Also, in severe cases, no findings in favor of Kaletra were observed compared to the control. Therefore, Kaletra alone is ineffective for Covid-19 and some medications should be added to Kaletra regimen like ribavirin and corticosteroid (18) or higher doses are needed to better demonstrate its effectiveness in prohibiting Covid-19 replication (19).

Among all patients, 96 were males, 47 were in the group 2 and 49 ones in the group1. Evaluation of hospitalization and age distribution did not show any specific findings. Of the 170 patients admitted, 88 were over 65 years, of whom 52 were in the group1. Also, of 40 patients under 50 years old, only 12 patients were in group1 (table 1). There was no difference between cut-off points of 5 days between two groups; 74 patients were discharged for less than 5 days (38 patients in group 2 versus 36 in group 1) and 96 patients were discharged after 5 days (47 patients in group 2 versus 49 in the group1). Comparison of mortality and co-morbidities did not show a significant association (table 2). According to a study by Rahmani H et al.(20), DM and HTN are the most common co-morbidities in Covid-19. 27 patients with diabetics (21 patients in group 2 versus 6 in group1) and 86 cases of HTN (62 cases in the group1 and 24 cases in the group2) (table 1). With these co-morbidities distribution, the mortality was only 4 cases higher in group 1. Also, 52 patients in group 1 were over 65 years compared to 36 in group 2 (table 1). Chronic diseases such as cardiovascular and DM(21) are associated with a higher mortality by weakening patients' immune system and establishing inflammatory conditions (22). In group 1, 49 patients were discharged after 5 days while 12 patients died. Among the patients who died; HTN, DLP, and IHD were more common. Also among the 12 patients who died after 5 days of hospitalization; two third of patients were over 65 years old and 10 of them were males. Although, 72.9% had HTN (vs 28.2% in the ATV group), it could not impact on mortality. Therefore, most patients had HTN and more than half of them were over 65 years, but there was no significant difference in mortality compared to the group 2 (tables 1 and 2).

Despite Kaletra being the first choice of therapeutic regimen for Covid-19 cases (23), ATV is another PI agent that is safer than LPV in HIV patients.(4, 24) Besides, it is much more potent than LPV and Kaletra (25). The mechanism of its action is binding affinity. It has a potential to bind different parts of coronavirus encompassed viral enzymes(such as helicase and ribonucleic acid (RNA) polymerase) and replication complex (26). 

In one study, Bo Ram Beck et al. evaluated the binding affinity of antiviral agents using the molecule transformer-drug target interaction (MT-DTI). Among the drugs, ATV, remdesivir, efavirenze, ritonavir, and dolutegravir were the most potent against 3C-like proteinase, respectively. In addition, ATV had the highest combined affinity for the replication complex. These findings suggest that ATV may be a good choice against Covid-19 (26). Interestingly enough, it has been demonstrated that ATV could access the lungs via intravenous administration (27, 28). Also, it could reach to the lungs even in pulmonary fibrosis (28). Natalia Fintelman-Rodrigues et al. (25) reported the same results of experimental studies about lung bioavailability and potency of ATV.(27, 29) Natalia Fintelman-Rodrigues et al.(25) indicated that the severity and cell death of Covid-19 had direct association with lactate dehydrogenase (LDH) levels and also serum interleukin 6 (IL-6). ATV could reduce IL-6 levels in Covid-19. They have also indicated similar efficacy of ATV in reducing levels of tumor necrosis factor-alpha (TNF-α). Their study has demonstrated that ATV and CQ have the same efficacy in in-vitro and superior to Kaletra. These features encouraged researchers to assess ATV efficacy.

Despite the 24.7% frequency of DM in group 2, analysis of discharge data for more than 5-days hospitalization showed similar result. Therefore, maybe the efficacy of ATV is more than Kaletra and is more potent in the treatment of Covid-19. In group 2, 47 patients were discharged after 5 days, of which 10 patients died. Among the 10 patients who died; HTN and HF were more common than other co-morbidities. The rest of the dead patients had no co-morbidities. About 70% of dead patients were males and 60% of them were over 65 years (tables 1 and 2). Comparison of the discharge data for less than 5 days hospitalization showed similar results and also did not show higher efficacy of Kaletra in the early stages of hospitalization unlike the previous study (18). A notable thing in our study was the 24.7% prevalence rate of DM (vs 7.1% in the Kaletra group) in the ATV group. DM is a poor prognostic factor for mortality in Covid-19 (30) but comparison of ATV to Kaletra group, we did not observe more mortality in the former group. Although, less than half of the patients were over 65 years and DM was much more common in group 2, fewer deaths were reported. Moreover, there were more dead patients in the group 1, but it was not statistically significant. Among all patients, male gender was considered as an important risk factor related to mortality (P=0.009) (tables 1 and 2). Our result is in line with Safiya Richardson’s study (31). Also, age over 65 years and HTN were the most prominent features of dead patients.

In a retrospective study of 213 patients with Covid-19 treated with ATV + HCQ, Hamid Rahmani et al.’s study compared the patients' prognosis based on O2 sat and pneumonia severity. Patients were divided into 2 groups: moderate form (110 patients) and severe form (103 patients). Comparison of hospitalization showed a higher discharge rate in the moderate group (77.27% versus 49.51% in the severe group). Also 28-days mortality assessment showed 6 fold lower for moderate group than severe one. Comparison of complications showed a higher rate in the severe group. This study supports the effectiveness of this combination regimen in patients with O2 sat of more than 90% and use the regimen as soon as possible to further benefits (20).

Diarrhea was the most common complication observed in group 1. Cao B et al. reported some adverse events among 46 patients on the Kaletra regimen on day 28, that GI complications including diarrhea, nausea, and vomiting were more common. Kaletra was also discontinued in 13.8% of patients. It is also noteworthy that liver and pancreatic enzymes should be monitored during treatment (18). In our study, nausea was the most common in the group 1, followed by diarrhea, epigastric pain, abdominal pain and vomiting, respectively. Also, an increase in LFTs was observed in only 3 patients of group 1. Complications like elevated liver enzymes, elevated indirect bilirubin, and liver injury have been attributed to the ATV administration (26). No evidence of GI complications was reported in any of the patients receiving the ATV regimen, but 3 patients of group 2 had a two- to three-fold increase in LFTs after receiving the regimen (table 3) . There was also reported evidence of jaundice and indirect hyperbilirubinemia in one patient with abnormal glucose-6-phosphate dehydrogenase deficiency (G6PD) level. It should be noted that about half of the patients in our study were over 65 years also more than half of them had at least one co-morbidity, so it is not possible to judge exactly the effectiveness of the combined regimens used and more studies are needed. As a result, although each group had known prognostic factors for Covid-19 (in group 1 significantly age over 65 years and a history of HTN and in group 2 significantly a history of DM), the comparison of mortality and hospitalization were not significantly different. Inconsistent results may be due to 1) impact of confounding factors on antiviral agents 2) small sample size 3) different disease stages 4) various sensitivities of endpoints 5) retrospective study. Therefore, RCTs with large sample size, more sensitive endpoints and patients with the same stages could show the efficacy and safety better. So far, this is the first study to compare the efficacy of Kaletra and ATV. 

In conclusion although, we did not find a significant difference between the efficacies of these two regimens, we prefer ATV because it tolerates better. Our results can be a good guide for clinicians considering the safety and efficacy of these antiviral agents but RCTs may determine the effectiveness of these two regimens better.
